# It’s Not Cool to Go Cold Turkey When Quitting Nicotine Chewing Gum: A Case Report

**DOI:** 10.7759/cureus.82566

**Published:** 2025-04-19

**Authors:** Ramya R Iyer

**Affiliations:** 1 Public Health Dentistry, Sumandeep Vidyapeeth, K M Shah Dental College and Hospital, Vadodara, IND

**Keywords:** dependence, nicotine gum addiction, nicotine replacement therapy, pharmacotherapy, withdrawal symptoms

## Abstract

Past smokers under nicotine replacement therapy, such as nicotine gums, may develop an addiction to nicotine gums. This case report presents a case of nicotine gum addiction. The mother of a 28-year-old male presented at a private dental & tobacco cessation clinic with her son’s complaints of severe cravings after her son had reportedly gone cold turkey as an impulsive attempt to quit nicotine chewing gums. She reported that her son had a history of chewing >10 nicotine gums/day for the last three years. The mother reported that her son had been without nicotine for the last two days before he had agreed to seek professional help. She presented that her son had repeatedly failed to stop using nicotine gum use in the past. Detailed tobacco and nicotine gum history was recorded. Current nicotine dependence was assessed using a modified Fagerstrom scale for smokeless tobacco. The patient’s withdrawal symptoms were more of a psychological nature than disturbances of physiology. As per the patient’s preference, tele-counseling sessions were conducted daily. Additionally, supportive and interventional measures were recommended and reinforced through WhatsApp messages. As there were rebounds of anxiety attacks, in consultation with a psychiatrist, after a week of counseling, pharmacological management was included as an adjunct to help improve the patient’s confidence. The patient had abstained from nicotine gums for one complete month from the date of reporting, with only negligible withdrawal symptoms. A one-month follow-up of the patient confirmed no signs of relapse. The report brings to light that dealing with going cold turkey in long-term nicotine gum addiction can be challenging and recommends a planned professional cessation therapy in order to minimize withdrawal symptoms.

## Introduction

Nicotine gums are over-the-counter (OTC) products for tobacco cessation (nicotine replacement therapy). There are high risks of these products being misused, and they pose a potential threat of dependence. Past smokers who were under nicotine replacement therapy, such as nicotine gums, can develop an addiction to nicotine gum, although reported as a rare consequence in the literature [1].

In an analysis of actual purchase patterns in a population-based sample in the US, Shiffman et al. [2] reported that persistent use of nicotine gum and patches was very rare (1%) and had not increased with the transition to OTC use despite the removal of physician oversight.

Mulry [3] reported in his work titled, "Nicotine gums-a positive addiction" implied no or minimal adverse effects related to the use of nicotine chewing gums. However, contradictory findings have been reported in the literature related to safety concerns of nicotine chewing gums. A study by West et al. [4] showed that nicotine gum could produce severe withdrawal symptoms, such as increased irritability, difficulty in concentrating and a drop in the heart rate, when the addicted abstained from nicotine gum. According to Eliason et al. [5], long-term nicotine gum use resulted in insulin resistance and hyperinsulinemia. A negative relationship was observed between insulin sensitivity and cotinine levels. Lee et al. [6], in a recent clinical case report, presented a case of fatal nicotine gum toxicity resulting in a respiratory emergency.

There is a limited description of protocols for the management of nicotine chewing gum de-addiction in the literature. Hurt et al. [7] recommended the following strategies as equally effective for cessation of long-term nicotine gum use: (a) abrupt cessation, (b) taper with placebo gum (marginally increased chances of relapse with the tapering with placebo gum), and (c) taper with nicotine gum. 

Cessation of nicotine gums can be challenging when not guided or assisted by an appropriate healthcare professional. Abrupt cessation without any professional assistance is called as going "cold turkey". It is based on the assumption that people work with willpower to quit the adverse habit at once. Going cold turkey is an unjustified oversimplified solution to quit nicotine all of a sudden with a high risk of severe withdrawal symptoms.

This case report presents an exploratory approach to study an underreported but important issue of nicotine gum addiction. This helps to give visibility to the potential misuse of over-the-counter (OTC) nicotine therapy.

## Case presentation

The mother of a 28-year-old male entrepreneur visited a private dental and tobacco cessation clinic in Vadodara, Gujarat, India. She presented her son’s complaints of severe cravings after he had reportedly gone cold turkey as an impulsive attempt to quit nicotine chewing gums. She reported that her son had a history of chewing 11-14 nicotine (2 mg) gums/day for the last three years (2020-2023). The mother reported that her son had been without nicotine for the last two days before he had agreed to seek professional help. She presented that her son had repeatedly failed to stop using nicotine gum use in the past. In patient’s own words, the withdrawal symptoms that were reported were “anxiety,” “gloominess,” “negative thinking,” and “over-thinking,” which were clearly psychological rather than disturbances of physiology.

The patient had a past history of smoking cigarettes (approx. 12/day) from 2012 to 2020 (from the age of 17 years). He also had a history of vaping, while at Hong Kong from 2015 to 2020. It was reported that the patient had quit all forms of tobacco after 2020. The patient reported a history of medication. His family physician and psychiatrist had put him on Zolfresh 10 mg (Zolpidem Tartrate), a prescription medicine for treating insomnia, for the last three months.

At the outset of the patient management, the patient’s permission was obtained to use the details of the patient’s experiences and WhatsApp interactions for sharing and dissemination for academic/educational purposes. An assurance of anonymity was given to the patient, and written informed consent was taken from the patient for the present case report. At the baseline, nicotine dependence (from nicotine gum) was assessed using the Modified Fagerstrom Scale for Smokeless Tobacco [8]. The nicotine de-addiction strategy was based on the Ministry of Health and Family Welfare (Government of India) Tobacco Dependence Treatment Guidelines [9].

As per the patient’s preference, tele-counseling sessions were conducted daily. Patients’ compliance was ascertained, and the caretaker’s feedback was obtained.

**Figure 1:**
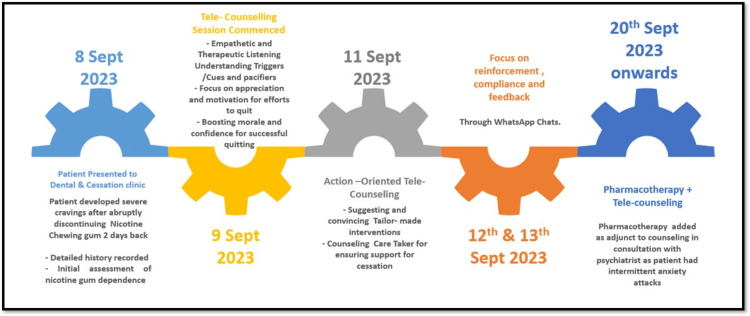
Timeline of tailor-made interventions for nicotine gum cessation.

As there were rebounds of anxiety attacks, and the patient was on medication for insomnia, in consultation with a psychiatrist, after 12 days of counseling, pharmacological management was included as an adjunct to help improve the patient’s confidence. The patient had been prescribed Bupropion (Bupron SR 150 mg/day for the first week, followed by Bupron SR 300 mg/day) by his family physician and psychiatrist. The timeline detailing the various steps in nicotine gum cessation intervention is depicted in Figure 1.

**Figure 2:**
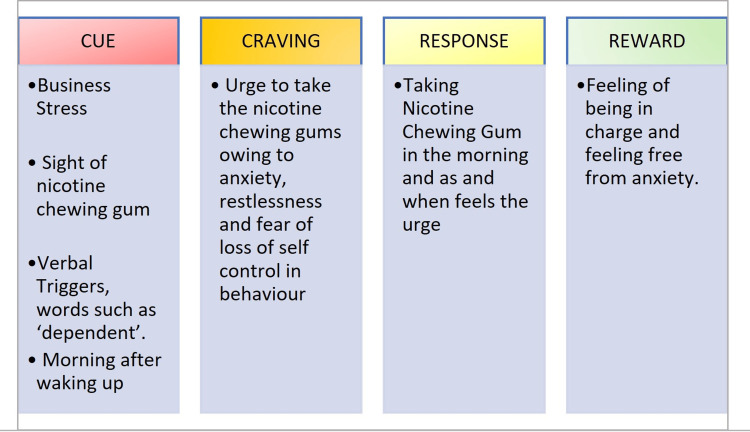
Patient's habit loop components.

**Figure 3:**
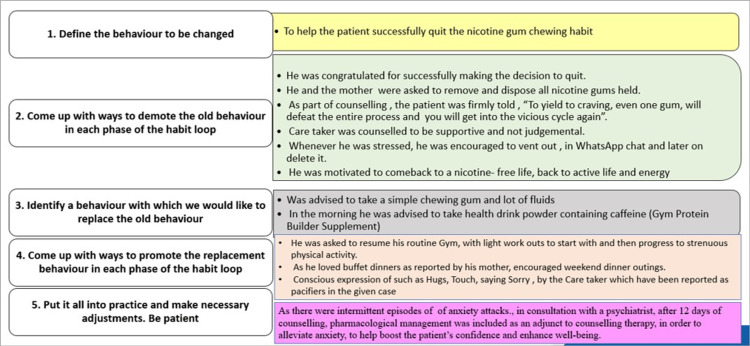
Stage/level-wise targeting of the habit loop for behavior modification.

The habit loop approach, an evidence-based method in the areas of behavior psychology and behavior modification, was applied in the present case. Positive behavioral change was attempted by identifying the components of the level of the habit loop, i.e., cue, craving, response, and reward [10]. After identifying the components of cues, cravings, response, and reward, specific interventions were designed to suit the patient in a tailor-made fashion to disrupt the adverse habit loop and create a new favorable habit loop. The identified components of the habit loop for the patient and the detailed measures targeting each level of the existing adverse habit loop are depicted in Figures 2, 3.

Feedback on tele-counseling and ascertainment of compliance through WhatsApp with a family member (mother of the patient) was noted, and timely suggestions were made to the course of care.

**Figure 4:**
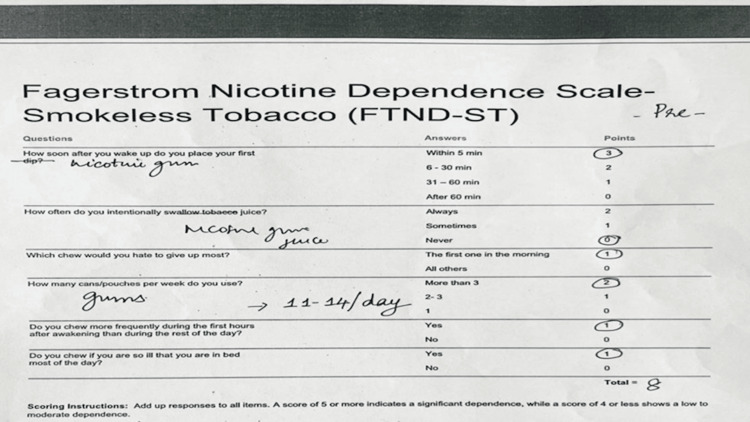
Baseline FTND-ST score. FTND-ST: Fagerström Test for Nicotine Dependence-Smokeless Tobacco.

**Figure 5:**
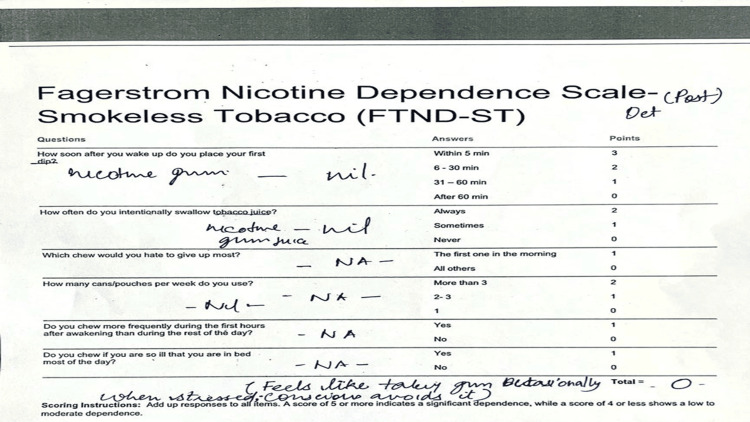
FTND-ST scale score at one-month follow-up. FTND-ST: Fagerström Test for Nicotine Dependence-Smokeless Tobacco.

On the first follow-up, on the 10th of October 2023 (after one complete month from the date of reporting), it was observed that the patient had abstained from nicotine gums. The patient reported had negligible withdrawal symptoms on the 10th of October 2023 [8]. A change in nicotine dependence score from high dependence (score 8) at baseline to no or negligible dependence (score 0) on one-month follow-up was observed (see Appendix, Figures 4, 5).

The patient was followed up once a month for a period of one year. On a one-year follow-up on the 17th of October 2024, the patient confirmed complete abstinence from nicotine gums and the absence of any associated symptoms.

## Discussion

Long-term use of nicotine-containing chewing gum is associated with chronic and acute adverse effects, which suggests that the use of nicotine replacement therapy during smoking cessation should be monitored. There is a need to manage the patients with nicotine gum addiction, as in the present case, in a strategic and tailor-made fashion.

In the present case, a combination of counseling and pharmacotherapy was warranted as the patient’s withdrawal symptoms were majorly psychological in nature. A similar plan of management with a successful outcome was reported in case reports authored by Bhatia et al. [11] and Pekgor et al. [12]. Mendelsohn [13] observed that in patients with long-term nicotine addiction with physiological disturbances such as increased sweating and abdominal discomfort without major psychological manifestations, nicotine patches could be more useful. In a case of long-standing nicotine addiction reported by Agarwal et al. [14], adjunctive pharmacotherapy was advised as highly dependent patients might not respond to counseling as a stand-alone intervention.

In the present case, the patient had quit the nicotine gums all of a sudden (cold turkey) in spite of long-term use, which posed the challenges of keeping up patient motivation against physiological and psychological repulsions. Frequent bouts of anxiety during the intervention period led to the closely coordinated efforts of the counselor (trained public health dentist in tobacco cessation) and the family physician and psychologist in a framework of collaborative care.

The strength of this case report is that it is significant with respect to calling attention to a new challenge encountered in using nicotine replacement therapies. Tele-counseling was rendered in the case owing to the patient’s preferences; it is possible that in-person face-to-face counseling could have brought out more experiences and findings, both subjective and normative. Serum cotinine levels could not be estimated. In spite of the limitations mentioned above, the presented case management explicitly exemplified a treatment plan in a rarely reported scenario of nicotine gum addiction with a successful outcome.

## Conclusions

Although nicotine gum addiction has been reported as a rare case, it deserves attention and there is a need to develop a definitive treatment and management protocol. Dealing with going cold turkey in long-term nicotine gum addiction can be challenging owing to high nicotine dependence. It may be appropriate to seek professional cessation counseling and therapy for nicotine gum de-addiction rather than going cold turkey. Research should be directed towards correlating cotinine levels in patients with prolonged self-medication with nicotine chewing gum. It is also recommended that nicotine gums, which are OTC products, need to be regulated, and use of prolonged usage must be documented and reported by a pharmacist.
